# A Combined Digital PCR and Next Generation DNA-Sequencing Based Approach for Tracking Nearshore Pollutant Dynamics Along the Southwest United States/Mexico Border

**DOI:** 10.3389/fmicb.2021.674214

**Published:** 2021-08-06

**Authors:** Amity G. Zimmer-Faust, Joshua A. Steele, Xianyi Xiong, Christopher Staley, Madison Griffith, Michael J. Sadowsky, Margarita Diaz, John F. Griffith

**Affiliations:** ^1^Southern California Coastal Water Research Project, Costa Mesa, CA, United States; ^2^BioTechnology Institute, University of Minnesota Twin Cities, Saint Paul, MN, United States; ^3^Department of Soil, Water, and Climate, University of Minnesota Twin Cities, Saint Paul, MN, United States; ^4^Proyecto Fronterizo de Educación Ambiental, A.C., Tijuana, Mexico

**Keywords:** microbial source tracking, 16S ribosomal DNA analysis, wastewater, droplet digital PCR, coastal water

## Abstract

Ocean currents, multiple fecal bacteria input sources, and jurisdictional boundaries can complicate pollution source tracking and associated mitigation and management efforts within the nearshore coastal environment. In this study, multiple microbial source tracking tools were employed to characterize the impact and reach of an ocean wastewater treatment facility discharge in Mexico northward along the coast and across the Southwest United States- Mexico Border. Water samples were evaluated for fecal indicator bacteria (FIB), *Enterococcus* by culture-based methods, and human-associated genetic marker (HF183) and *Enterococcus* by droplet digital polymerase chain reaction (ddPCR). In addition, 16S rRNA gene sequence analysis was performed and the SourceTracker algorithm was used to characterize the bacterial community of the wastewater treatment plume and its contribution to beach waters. Sampling dates were chosen based on ocean conditions associated with northern currents. Evidence of a gradient in human fecal pollution that extended north from the wastewater discharge across the United States/Mexico border from the point source was observed using human-associated genetic markers and microbial community analysis. The spatial extent of fecal contamination observed was largely dependent on swell and ocean conditions. These findings demonstrate the utility of a combination of molecular tools for understanding and tracking specific pollutant sources in dynamic coastal water environments.

## Introduction

Once bacterial and chemical contaminants enter the nearshore coastal environment, complicated mixing, dilution, and transport processes make it increasingly difficult to identify their origin. Moreover, jurisdictional boundaries, including international borders, can further complicate investigations aimed at identifying specific sources of contamination along coastlines. Despite this, fecal pollution represents a leading cause of water quality impairments in coastal waters worldwide, and it is critical to identify its origin for successful management and mitigation of associated public health and economic consequences (DeFlorio-Barker et al., 2018).

In recent years, microbial source tracking (MST) methods that differentiate amongst different specific animal and human fecal sources have been developed and their application has become more widespread. Non-human sources of FIB do not carry the same pathogenic load compared to human point sources such as sewage (Soller et al., 2010, 2014; Schoen et al., 2011), underscoring the need to understand where fecal contamination is coming from. Among the different MST-based approaches, the marker gene approach, which relies on measurement of host source-associated DNA sequences by polymerase chain reaction (PCR)-based technologies, is among the most utilized (Harwood et al., 2014). Source-associated markers have been developed for common fecal sources including cow (Shanks et al., 2006, 2008; Kildare et al., 2007), dog (Green et al., 2014b), bird (Shanks et al., 2008; Lee et al., 2013), and human (Seurinck et al., 2005; Green et al., 2014a). Recent advances in digital PCR provide enhanced sensitivity and robustness to inhibitory substances (Cao et al., 2015), with select quantitative PCR (qPCR)-based MST assays readily adapted to digital PCR (Cao et al., 2015; Coudray-Meunier et al., 2015; Staley Z. R. et al., 2018). The MST marker gene approach has been used previously to effectively identify and differentiate between specific fecal sources in a variety of matrices, including estuarine (Riedel et al., 2015), fresh waters (Li et al., 2019), marine waters (Ervin et al., 2013), and sediments (Zimmer-Faust et al., 2017), resulting in recommendations for application of best management practices, specific infrastructure improvements, and regulatory support (USEPA, 2011; Verhougstraete et al., 2015; Goodwin et al., 2017).

More recently, technological advances in next generation sequencing (NGS) technology have led to source identification based on the comparative characterization of the entire microbial communities of environmental samples and pollution sources (Ahmed et al., 2015; Roguet et al., 2018; Staley C. et al., 2018), providing an additional avenue for identifying the origins of fecal contamination. These methods differ from the single marker MST-based approaches by their ability to characterize thousands of sequences in each sample (Sogin et al., 2006), in theory making them able to characterize any relevant source. Computational tools utilizing sequencing data that allow for community-based microbial source tracking have been developed and include the SourceTracker program (Mathai et al., 2020; Roguet et al., 2020). The SourceTracker program uses a Bayesian algorithm to estimate the proportion of each source in a set of samples (Knights et al., 2013). Previous studies have successfully applied the SourceTracker program to characterize bacterial contamination sources in recreational fresh (Baral et al., 2018) and estuarine (McCarthy et al., 2017) waters, with success of these methods reliant on microbial community profiles that are unique to each source (Brown et al., 2017; Staley C. et al., 2018). However, application of these MST tools for tracking specific pollutant sources within the nearshore coastal environment has not been explored fully, with previous efforts primarily focused on identifying the source of pollution to impacted water and ignoring the geographic reach of contamination. McCarthy et al. (2017) used microbial community-based source tracking to validate a 3-dimensional estuarine hydrodynamic model, finding this approach successful at identifying the primary water sources contributing to an urban estuary. However, in the nearshore coastal environment, mixing is further complicated by surf zone processes that can impact pollutant transport significantly.

In this study, the extent to which effluent discharged by a wastewater treatment plant in Tijuana, Mexico is transported within the nearshore coastal environment was examined. It has been hypothesized that when ocean currents are flowing south-to-north, wastewater from the outfall of the San Antonio de los Buenos (SADB) wastewater treatment plant (WTP) at Punta Bandera in Tijuana, Mexico contributes to elevated bacterial levels and introduces human fecal contamination to nearshore waters from its origin, across the United States/Mexico border, and as far north as the City of San Diego, CA, United States (Orozco-Borbón et al., 2006; Sassoubre et al., 2012; Thulsiraj et al., 2017). Previous spatial modeling efforts have suggested that south-to-north coastal currents have the potential to transport discharges from the SADB plant and impact water quality in southern San Diego County (CH2M Hill, 2009; Feddersen et al., 2020). However, the extent and impact of the plume has never been verified, in part due to logistical challenges associated with sampling across an international border. In this study, a combination of MST technologies consisting of human-associated marker genes and NGS was used to detect, quantify, and track human fecal contamination emanating from the outfall of the SABD WTP during specific south-to-north swell conditions from its origin in Tijuana, Mexico to San Diego, CA, United States.

## Materials and Methods

### Study Site

**FIGURE 1:**
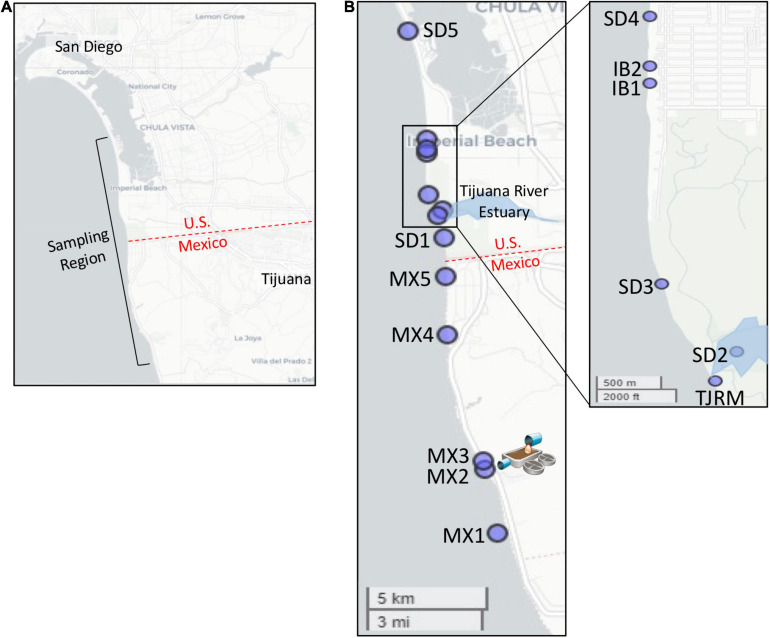
Map of sampling locations. **(A)** Sampling region targeted. **(B)** Location of specific sites sampled. Map of Imperial Beach region inset. SAB WTP outfall located at site MX2.

The Southwest United States border region has a long history of poor water quality, with elevated levels of fecal indicator bacteria (FIB) leading to frequent beach postings and closures. At popular surf beaches near the United States/Mexico border in San Diego, CA, United States, sources of FIB causing poor beach water quality are unknown and of concern to public health officials, given the proximity of multiple potential inputs of human fecal contamination, which include the outfall of the SADB WTP in Tijuana, Mexico (Kim et al., 2009). The SADB WTP has capacity to treat up to 35 million gallons per day (MGD) and receives a mixture of wastewater from the cities of Tijuana and Rosarito, Mexico (USEPA, 2014). The plant is located approximately 7.7 km south of the United States/Mexico border and discharges minimally treated effluent to a coastal stream that terminates at the ocean at Punta Bandera, approximately 5 km south of the treatment plant and 11 km south of the United States/Mexico border (Figure 1). During events when swells are coming from the south, the SADB source generally travels at 8–14 km/day (Feddersen et al., 2020), suggesting that the plume can reach the San Diego, CA, United States beaches targeted within this study within a 24–48 h period.

A second potential source of human fecal contamination in the region is the Tijuana River, which terminates into the Tijuana River estuary. The Tijuana River is 193 km long, flowing north through Mexico, before entering the United States. During dry weather, flows in the Tijuana River are largely intercepted by diversion structures in Mexico and pumped to either the SADB WTP or the South Bay International WTP before they cross into the United States. At times, however, pumps in the diversion structures can become clogged or malfunction, allowing potentially contaminated water to flow across the border into the United States (USEPA, 2014).

In the present study, sampling was designed to characterize impacts specifically from the SADB WTP. An approximately 23 km stretch of coastline was targeted, extending from just south of the SADB WTP discharge at Punta Bandera, Mexico to Silver Strand State Beach, CA, United States. Sampling took place during dry weather, to reduce the expected impact from the Tijuana River and other runoff-related sources. South swells dominate the coastal ocean in this region during the dry season and specific south swell events were targeted for sampling to increase the likelihood of capturing a gradient of human fecal pollution extending north from the SADB WTP outfall.

### Sample Collection

Sites were sampled during dry weather along the coastline from just south of the SADB WTP outfall at Punta Bandera to Silver Strand State Beach, CA, United States (Figure 1 and Table 1). Four dry weather, south swell events were targeted for sampling between October 2018 and October 2019. Two to three consecutive days were sampled per south swell event (Supplementary Table 1).

**TABLE 1:** Description of sampling locations.

Site ID	Site	Latitude	Longitude	Distance to SADB WTP outfall	Description
MX1	SADB South	32.43786	−117.1019	1.2 km (South)	San Antonio del Mar, South of SADB WTP outfall; Ocean Site
MX2	SADB WTP Discharge	32.44804	−117.1053		SADB WTP outfall at Punta Bandera
MX3	SADB North	32.447652	−117.1086	0.3 km	North of SADB WTP (mixing zone); Ocean Site
MX4	El Vigia	32.502769	−117.1236	6.3 km	Southern end of Playas; Ocean site
MX5	Parque Mexico	32.527417	−117.1245	9.0 km	Parque Mexico; Ocean Site
SD1	Border field	32.5434	−117.125	10.8 km	Border Field State Park; Ocean site
SD2	TJ Slough	32.55299	−117.128	12.6 km	Tijuana River Estuary; Estuary site
TJRM	TJ River Mouth	32.55235	−117.127	11.7 km	Tijuana River Mouth; mixing zone
SD3	TJ Slough beach	32.561	−117.132	12.9 km	Tijuana Slough National Wildlife Refuge; Ocean site
IB1	Elder Ave	32.5788	−117.133	14.8 km	Imperial Beach; Elder Ave, Ocean site
IB2	Elm Ave	32.5803	−117.133	15.0 km	Imperial Beach Pier; Elm Ave, Ocean Site
SD4	Carnation	32.5847	−117.133	15.48 km	Imperial Beach municipal beach; Carnation Ave, Ocean Site
SD5	Silver Strand	32.6296	−117.141	20.4 km	Silver Strand State Beach; Ocean Site

Grab water samples (2 L) were collected from each site. At all beach sites, ankle-to-knee deep water (swash zone) samples were taken on each sampling date. At site SD2, located just inside the mouth of the Tijuana River Estuary, water was collected with a pole sampler just below the water surface.

In addition, paired surf zone samples (approximately 100 m offshore) were also collected from personal watercraft at all beach sites north of the United States/Mexico border (*n* = 7) during two of the four south swell events (Event 3 and Event 4). Surf zone samples were collected on all 3 days during Event 3 and during the first 2 days of Event 4 (Supplementary Table 1). Grab water samples were also collected from the two potential sources: the SADB WTP outfall at Punta Bandera (site MX2) and upstream in the Tijuana River, before it crosses into the United States. Samples were collected from the SADB WTP outfall on each sampling date to characterize effluent quality. Due to logistical challenges associated with access to the Tijuana River, upstream of the United States/Mexico border and the diversion structures, four grab samples total were collected on two sampling dates (August 1, 2019 and August 9, 2019). These samples were collected to characterize potential inputs to the Tijuana River that are theoretically diverted during dry weather.

All environmental sites were sampled in the morning to limit degradation of the bacterial signal. Water samples were stored on ice and transported to either laboratories at the Southern California Coastal Water Research Project (SCCWRP) in Costa Mesa, CA, United States (if collected north of the United States/Mexico border) or to laboratories at the Proyecto Fronterizo de Educación Ambiental, A.C. in Tijuana, Mexico (if collected south of the United States/Mexico border) for sample processing, which included analyzing for culturable enterococci and filtering for bacterial DNA. Filters were then transported to SCCWRP laboratories for analysis by digital PCR.

The four sampling events differed in swell direction and magnitude and tidal and wind conditions. Conditions per event are summarized in more detail in the Supplementary Material (Supplementary Figures 1–4). During the first sampling event (Event 1), samples were collected on two consecutive days with swell direction primarily from the southwest (Supplementary Figure 1). During events 2 and 3, similar conditions were sampled- the beginning of a south swell event. Samples were collected on three consecutive days with swell direction primarily from the west on Day 1 and from the south on Days 2 and 3 (Supplementary Figures 2, 3). During event 4, the end of a south swell was targeted. Samples were collected on three consecutive days, with swell direction switching to more mixed by day 3 (Supplementary Figure 4).

### Sample Processing

#### FIB Cultivation

Cultivable *Enterococcus* was quantified by using Enterolert in conjunction with the Quanti-Tray 2000^TM^ system (IDEXX, Westbrook, ME, United States), as per the manufacturer’s instructions. Quantification was done using three dilutions covering a 100,000-fold range of concentration. Field and equipment blanks were collected and tested for FIB contamination in the same manner as regular samples. Laboratory blanks were performed using sterile phosphate buffered saline solution.

#### Filtration for Bacterial DNA

Fifty to 250 mL aliquots of sample water were filtered through 47 mm, 0.4 μm pore size, HTTP polycarbonate filters (EMD Millipore, Billerica, MA, United States). Each filter was placed in an individual 2 mL polypropylene screw cap tube, containing 0.7 mL dry volume of ZR BashingBead lysis matrix high density beads and 1 mL DNA/RNA Shield solution (Zymo Research, Costa Mesa, CA, United States). Bead tubes were stored at 4°C until transport to SCCWRP, and then stored at −80°C until processed. Filter blanks, consisting of sterile phosphate buffered saline passed through the polycarbonate filter, were also generated with each set of processed samples.

#### Microbial Source Tracking Marker Analysis

DNA was recovered from filters using the ZymoBiomics DNA Kit (Zymo Research, Costa Mesa, CA, United States), according to manufacturer’s guidelines. Extracted DNA was eluted into 100 μL of buffer and aliquots were stored at −20°C until analyzed with droplet digital PCR (ddPCR) on the Bio-Rad QX200 platform (Bio-Rad, Hercules, CA, United States). DNA from a halophilic, alkaliphilic archaeon (*Natronomonas pharaonis*) was added to the lysis buffer prior to extraction as an external extraction and inhibition control following USEPA method 1611 guidelines (USEPA, 2012). Negative extraction controls (NEC) containing only lysis buffer and halophile DNA were processed alongside each set of extracted samples.

Human-associated genetic markers and *Enterococcus* were quantified using digital PCR. Primer and probe sequences were those from published methods for detection of the HF183 (Cao et al., 2015) and Lachno3 (Feng et al., 2018) human-associated markers and for detection of *Enterococcus* (Cao et al., 2015). Primer and probe sequences are included in the Supplementary Material (Supplementary Table 2) and detailed methods employed have been described previously (Steele et al., 2018).

#### 16S rRNA Gene Sequence Analysis

Next-generation DNA sequencing was used to characterize microbial community structure at coastal sites along the United States/Mexico border region and at sites potentially contributing non-indigenous bacteria to coastal waters. The DNeasy PowerSoil Pro DNA extraction kit (QIAGEN, Hilden, Germany) was used to extract DNA from water filters for next generation sequencing, according to manufacturer’s guidelines.

The V5 and V6 regions of the bacterial 16S rRNA gene were amplified using primers F784 (5′-RGGATTAGATACCC-3′) and 1046R (5′-CGACRRCCATGCANCACCT-3′ (Claesson et al., 2010). Subsequently, the amplicons were pair-ended sequenced on the Illumina MiSeq platform (San Diego, CA, United States) using the dual-index method (Gohl et al., 2016) at the University of Minnesota Genomics Center (UMGC, Minneapolis, MN, United States).

Demultiplexed reads were analyzed via two different bioinformatic pipelines. The first was an OTU clustering-based approach and the second employed DADA2. For the OTU-based approach, the mothur pipeline v1.45.1 (Schloss et al., 2009) was utilized. Sequences were aligned against the SILVA Ribosomal RNA database v.132 and subjected to a 2% pre-cluster to remove likely sequence errors (Huse et al., 2010). Chimeric sequences were identified and removed using UCHIME ver. 4.2.40 (Edgar et al., 2011). Within each sample, operational taxonomic units (OTUs) were binned at 97% sequence similarity using the furthest-neighbor algorithm and were classified using the Ribosomal Database Project database v16 (Cole et al., 2009). For the DADA2 approach (Callahan et al., 2016), error rates were calculated and used for further quality filtering. Chimeras were removed using the removeChimeraDenovo function and taxonomy was assigned using DADA2’s RDP Bayesian classifier against the Silva v.132 database (Yilmaz et al., 2014; Glöckner et al., 2017). Sequence data was submitted to the NCBI SRA database with accession number SRP250574.

#### SourceTracker Analysis

SourceTracker (ver. 0.9.8) was used to assign sources of fecal bacterial contamination at all 11 sink sites in Figure 1 using default parameters in Knights et al. (2013). Separate SourceTracker assignment was run for each day to identify the compositional similarity in bacterial communities between sink sites and contamination “sources.” The source assignment was completed for duplicate samples from each site for each sampling date, with source assignments (%) averaged.

SourceTracker was run two ways for each site, which differed based on assignment of the contamination sources. SourceTracker was run initially with the following two sources categorized as follows:

(1) Pristine/marine: composition calculated from samples collected in Silver Strand Beach, CA, United States on days when the plume was not thought to impact sites this far to the north (*n* = 6: Event 1 Day 1 and Day 2, Event 2 Day 1, Event 3 Day 1 and Day 2, Event 4 Day 1).
(2) SADB WTP outfall site: composition calculated by using samples collected from the SADB WTP outfall at Punta Bandera (site MX2) on the same day as the sink samples.

In addition, SourceTracker was re-run and potential inputs to the upstream Tijuana River were included as a third potential source.

(3) Tijuana River: composition calculated using samples collected from upstream on the Tijuana River, before the diversion structures. This source was included as a proxy for potential inputs to the Tijuana River that are theoretically diverted during dry weather but may be making it across the border, into the Tijuana River Estuary, and contributing to water quality impacts at beach sites.

#### Additional Measurements

Salinity and water temperature were measured at all sites using a portable meter (YSI Model Pro30). Swell direction, wave height, and tidal cycle information were downloaded from publicly available databases (swell direction/wave height; tidal conditions)^1^ ^,2^ for all sampling periods.

### Statistical Analyses

All statistical analyses were done using R-studio (R-studio version 1.1.463, Boston, MA, United States, R version 4.0.1). Correlation tests were completed to compare indicator measurements (HF183, Lachno3, and *Enterococcus* by ddPCR and culture) to each other and to SourceTracker analysis source assignments. Multiple linear regression models were run comparing the relationship between human marker measurements (Lachno3 and HF183) and distance from the SADB outfall at Punta Bandera as a function of event differences.

For statistical analyses of microbial communities, samples were rarefied to a depth of 11,000 reads/sample. Differences between bacterial communities were visualized by principal coordinate analysis (PCoA) using Bray–Curtis dissimilarity matrices. Vegan (version 2.5.6) and phyloseq (version 1.32) packages were used for visualizing taxonomic trends.

## Results

### Human Fecal Marker and *Enterococcus* Alongshore Trends

**FIGURE 2:**
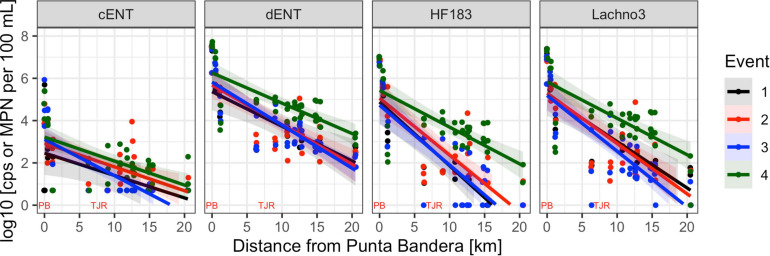
Enterococci by culture (cENT) and ddPCR (dENT) and human marker (HF183 and Lachno3) levels versus distance from the SADB WTP outfall at Punta Bandera. Each event is represented by a different color, with all dates sampled combined per event. On the *x*-axis location of the SSADB WTP outfall is noted (PB) as is the location of the Tijuana River Estuary (TJR).

Human-associated fecal marker (HF183 and Lachno3) and *Enterococcus* gene copies were evaluated in relation to increasing distance from the SADB WTP outfall at Punta Bandera (Table 2). There was a significant negative relationship between distance from the outfall and levels of human marker (HF183: *R*^2^ = −0.46, *p* < 0.01; Lachno3: *R*^2^ = −0.49, *p* < 0.01), evident during all four events (Figure 2). The slope of the relationship was significantly lower for Event 4 (*p* < 0.01), illustrating less dilution and likely faster transport of the plume when compared to Events 1–3. Estimated dilution of the SADB WTP plume was also calculated by comparing human-associated marker levels at each site relative to levels measured at the SADB WTP outfall, for estimated dilution see Supplementary Figure 5.

**TABLE 2:** Frequency of marker detection and concentration range detected for each marker by site.

Site	HF183			Lachno3			dENT			Frequency cENT > 104 MPN/100 mL
				
	Detection frequency	Concentration range		Detection frequency	Concentration range		Detection frequency	Concentration range		
		Lower	Upper		Lower	Upper		Lower	Upper	
MX1	100%	1.1E + 02	4.0E + 05	100%	3.2E + 02	5.0E + 05	100%	3.6E + 03	3.7E + 05	54%
MX2	100%	3.8E + 06	1.0E + 07	100.00%	5.4E + 05	2.5E + 07	100%	1.8E + 06	5.4E + 07	100%
MX3	100%	5.8E + 04	2.3E + 06	100.00%	8.5E + 04	6.3E + 06	100%	2.7E + 05	1.8E + 07	90%
MX4	82%	ND	1.2E + 04	90.90%	ND	3.0E + 04	100%	4.0E + 02	9.8E + 04	9%
MX5	89%	ND	2.6E + 04	100.00%	1.4E + 01	6.1E + 04	100%	4.5E + 02	1.3E + 05	11%
SD1	82%	ND	8.0E + 03	100.00%	4.3E + 01	2.0E + 04	100%	1.3E + 02	6.0E + 04	18%
SD2	60%	ND	2.2E + 04	100.00%	2.1E + 01	7.4E + 04	100%	5.4E + 02	1.1E + 05	20%
TJRM	67%	ND	8.4E + 03	100.00%	2.9E + 01	2.1E + 04	100%	8.0E + 02	7.4E + 04	33%
SD3	64%	ND	4.8E + 03	100.00%	1.8E + 01	1.5E + 04	100%	2.8E + 02	5.4E + 04	9%
IB1	83%	ND	7.5E + 03	100.00%	1.5E + 01	2.6E + 04	100%	3.3E + 02	9.2E + 04	17%
IB2	83%	ND	7.4E + 03	83.30%	ND	2.3E + 04	100%	5.5E + 02	8.4E + 04	0%
SD4	55%	ND	8.0E + 03	90.90%	ND	2.4E + 04	100%	1.1E + 02	7.9E + 04	0%
SD5	27%	ND	8.2E + 01	54.60%	ND	2.1E + 02	100%	6.4E + 01	2.5E + 03	9%

*For cultivable enterococci (cENT), frequency of exceedance of the single sample beach water quality public health threshold listed. Data combined across all four sampling events and sites are ordered from south to north.*

**FIGURE 3:**
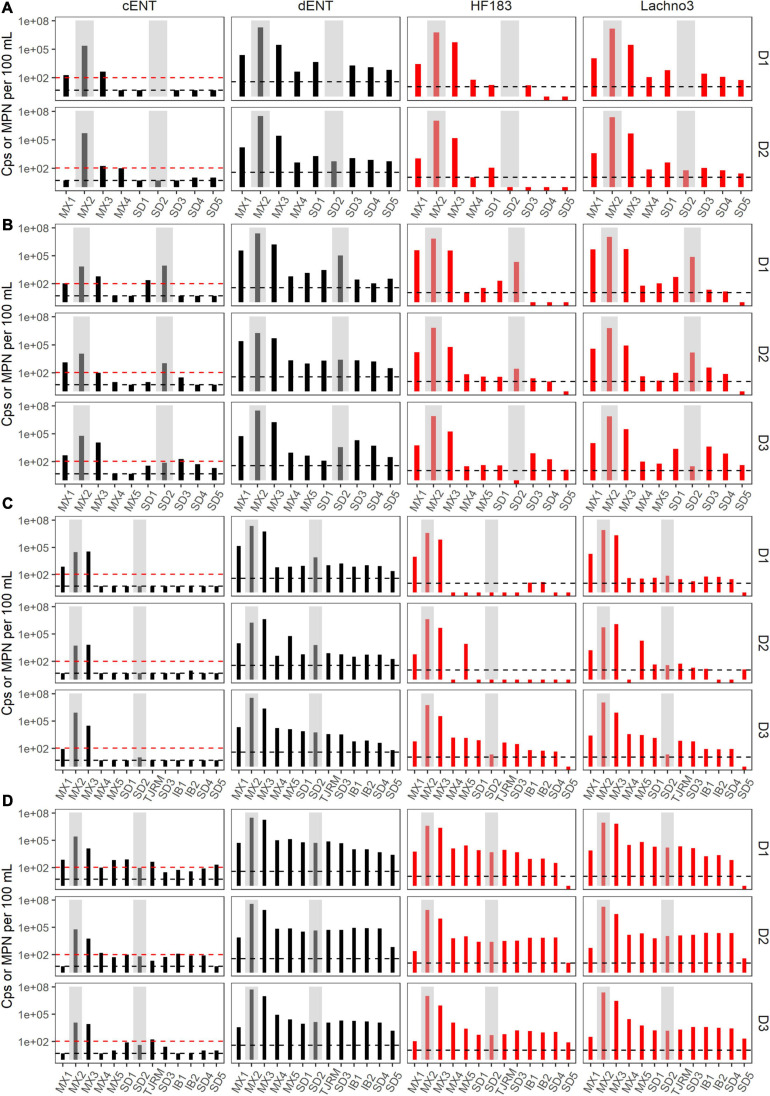
Enterococci (black bars) by culture (cENT) and ddPCR (dENT) and human marker (red bars), HF183 and Lachno3, results for Event 1–4 **(A–D)** for each day sampled (D1–D3). Gray bars are overlayed on top of sites MX2 (the SADB WTP outfall at Punta Bandera) and SD2 (the Tijuana River Estuary), representing the two non-beach sites. Black dashed lines represent the limit of detection and the red dashed line indicates the single sample beach water quality public health threshold for culturable enterococci of 104 MPN per 100 mL.

During Event 1, a gradient in human-associated marker levels was observed that extended to the United States/Mexico border. HF183 was not detected north of site IB1, while Lachno3 levels were detected at concentrations near the detection limit at the northernmost site (Silver Strand, SD5) on both sampling days. HF183 concentrations ranged from 547,250 copies/100 mL to not detected, with the highest concentrations at the sites bracketing the SADB WTP outfall at Punta Bandera (sites MX1 and MX3). Enterococci concentrations exceeded the single sample public health code regulatory limit (>104 MPN per 100 mL) at sites MX1 and MX3 on Day 1 and at Site MX3 (just North of Punta Bandera) on Day 2 only (Figure 3A).

During event 2, a sewage spill within a tributary of the Tijuana River confounded any along-coast human marker gradient observed. The spill bypassed the cross-border collector, leading to 0.86 million gallons of untreated sewage crossing from Mexico into the United States and being transported to the ocean via the Tijuana River Estuary the night before the first day of sampling. Water quality trends observed suggested that human marker detections were related to both the discharge from the SADB WTP outfall at Punta Bandera and the spill into the Tijuana River. Higher levels of both human markers were observed surrounding the two potential input sources, both bracketing the SADB WTP outfall at Punta Bandera and at sites located in the Tijuana River estuary (SD2) and just south of the terminus of the Tijuana River estuary (site SD1) (Figure 3B). Human marker levels decreased at sites near the Tijuana River Estuary by Day 3, likely due to dilution and degradation of the spilled sewage.

During Event 3, the swell switched from coming from the west to from the south by Day 3. Trends in human marker observed reflected the changing swell conditions, with a stronger south swell by Day 3 corresponding to an increasing gradient in human marker levels from the SADB WTP outfall moving toward the north. Human marker levels (HF183 and Lachno3) were similar on Day 1 and Day 2, with HF183 levels detected only at sites bracketing the SADB WTP outfall. By Day 3, human marker (HF183 and Lachno3) was detected extending from the SADB WTP outfall at Punta Bandera to Imperial Beach, CA, United States (sites IB1/IB2) (Figure 3). *Enterococcus* concentrations exceeded the single sample public health code regulatory limit (>104 MPN per 100 mL) at sites bracketing Punta Bandera on Day 1 and at the site just north of Punta Bandera (MX3) on Day 2 and Day 3. Otherwise, culturable *Enterococci* levels at all ocean sites were well below the single sample regulatory limit.

The strongest gradient in human marker levels occurred during Event 4, with quantifiable human marker levels observed at the site sampled farthest from the SADB WTP outfall, site SD5, by Day 3. Human marker levels were similar between sites MX5 and SD4, suggesting minimal dilution of the plume as it traveled north. Culturable enterococci concentrations exceeded, or were near the single sample regulatory limit of >104 MPN per 100 mL, at most sites between Punta Bandera, MX and San Diego, CA, United States on Day 1 and Day 2 of Event 4, with enterococci levels below the public health code regulatory limit at all sites, with the exception of the Tijuana River Estuary site (site SD2), on Day 3.

### Surf Zone Sampling

There were no significant differences observed between nearshore and surf zone *Enterococcus* by digital PCR and culture or human marker (HF183 and Lachno3) concentrations (all *p*-values > 0.05) (Supplementary Figure 7) from samples collected during events 3 and 4. In addition, measurements made in the surf zone and nearshore were highly correlated for all four targets measured (all *r*-values > 0.85; *p* < 0.01, Supplementary Figure 8).

### Contamination Source Determined by 16S rRNA and SourceTracker

The SourceTracker program has been previously validated in coastal waters using OTU clustered sequencing data (Henry et al., 2016; Staley C. et al., 2018). For consistency, the SourceTracker results discussed below reflect data processed using the OTU-clustering based approach. SourceTracker was also applied to ASVs (from DADA2). Results were compared, and compositional data was highly correlated using either method (*r* = 0.81, *p* < 0.01). Trends in SourceTracker defined contributions using DADA2 are included in the Supplementary Material (Supplementary Figure 6).

**FIGURE 4:**
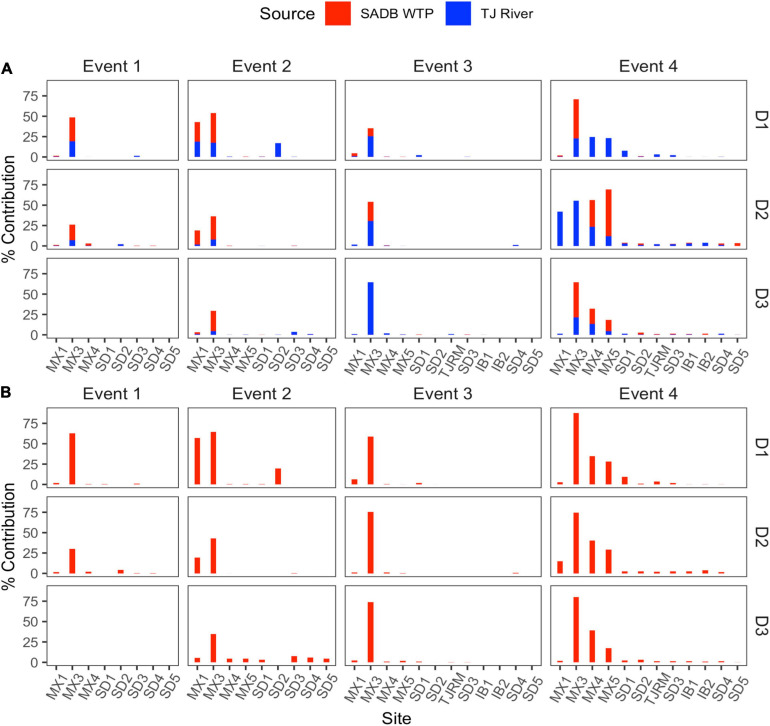
Contributions from potential sources (SADB WTP and Tijuana River) in each sink site using SourceTracker. Sites are arranged from a north to south along the *x*-axis. **(A)** SADB WTP included as the only source. **(B)** SADB WTP and Tijuana River included as potential sources.

Source assignment profiles differed by event, with results paralleling human-associated marker results when samples were evaluated with the SADB WTP as the only contamination source (Figure 4A). Estimated contributions of the SADB WTP outfall to beach sites north of the United States/Mexico border were highest during Event 4 with ∼3% of ocean water attributed to the SADB WTP. Although there was still evidence of impact from the SADB WTP plume to San Diego beach sites during the other events, total contributions to ocean waters were estimated to be much lower (<1%).

During Event 2, there was evidence of contamination contributed from the Tijuana River, which corresponded to the sewage spill event that occurred. When SourceTracker was run with both the SADB outfall and the Tijuana River included as potential sources, contributions were attributed to the Tijuana River at the Tijuana River Estuary site (site SD2) and at sites located adjacent to the estuary mouth (Figure 4B).

**FIGURE 5:**
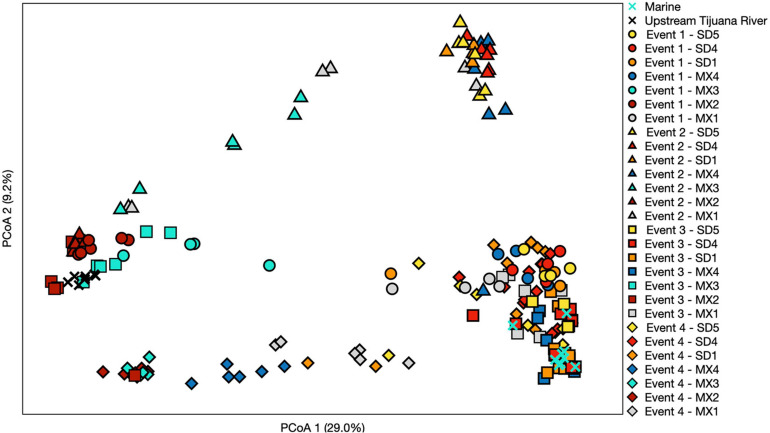
Principal coordinate analysis performed using a Bray–Curtis dissimilarity matrix. Each symbol represents a different sampling event, while each color represents a different site. Three sites north of the United States/Mexico border were chosen to include that represent trends observed. Numbers in parentheses denote % of variation explained by each axis.

Samples generally clustered by graphical location, with differences noted between events (Figure 5). Samples collected at the SADB WTP outfall at Punta Bandera, clustered together regardless of when they were collected, suggesting a homogeneous microbial community in the discharge. In contrast, samples collected from SD2 (the Tijuana River Estuary site), varied dependent on Event, clustering more closely to the SADB WTP outfall during Event 2, when there was a sewage spill into the Tijuana River the night preceding sampling. The beach samples clustered most closely to MX2 (the SADB WTP outfall site) during Event 4.

The geographic extent of the SADB WTP plume was also evidenced by the presence of abundant taxa associated with the SADB WTP plume in marine water samples. ASVs (from DADA2) associated with the SADB WTP plume were resolved to a lower taxonomic level (genus or species) when compared to OTUs; thus, taxonomic trends described below reflect assignments made by the DADA2 pipeline.

**FIGURE 6:**
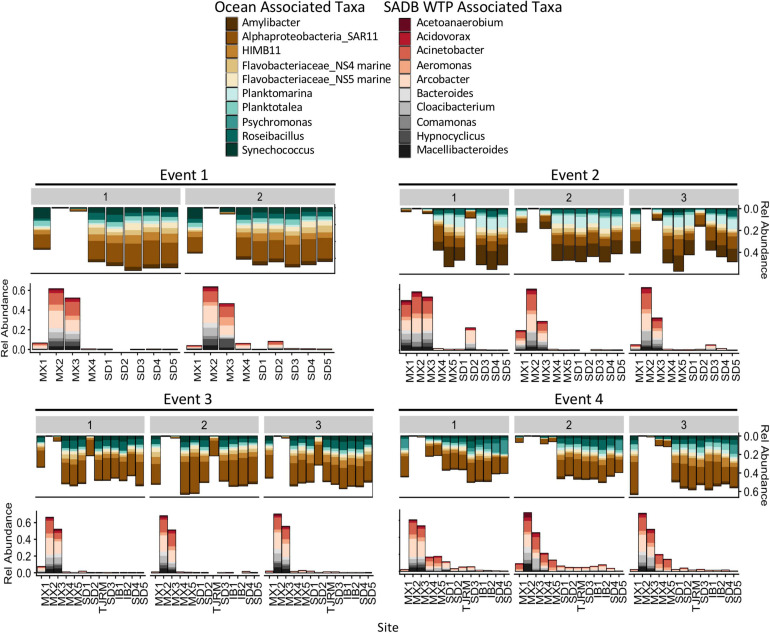
Relative abundances for the 10 most abundant families and genera associated with SADB WTP effluent stream (MX2) and Silver Strand (SD5) samples- representing the unimpacted marine microbial community. Relative abundances are shown at all sites moving south to north along the *x*-axis. Marine associated taxa are presented on the top and SADB WTP associated taxa are presented on the bottom.

Bacterial taxa that were dominant in the SADB WTP plume included several wastewater-associated genera, *Cloacibacterium*, *Comamonas*, *Acidovorax, Bacteroides*, and *Macellibacteroides*. There were also several potentially pathogenic genera commonly associated with wastewater that were present in high abundance including *Acinetobacter*, *Aeromonas*, and *Arcobacter*. Taxa associated with the SADB WTP (Site MX2) were generally present in decreasing abundances at sites moving to the north (Figure 6), with abundances dependent on event. The highest abundances of taxa associated with the SADB WTP were identified at beach sites during Event 4. During Event 2, high abundances of taxa associated with the SADB WTP were also observed at site SD2 (the mouth of the Tijuana River Estuary), reflecting the sewage spill event.

For comparison, abundant taxa associated with samples collected from Silver Strand State Park, CA, United States located 20 km north of the SADB WTP outfall at Punta Bandera, were used to characterize the unimpacted marine microbial community. Samples were evaluated from days when the plume was not thought to impact the Silver Strand State park site (*n* = 6: Event 1 Day 1 and Day 2, Event 2 Day 1, Event 3 Day 1 and Day 2, Event 4 Day 1). Marine-associated taxa were present in high abundance in those samples and included members of the marine Rhodobacteraceae family (*Amylibacter*, *Planktomarina*, *Planktotalea*, and from the coastal HIMB11 *Roseobacter* group), members of the SAR11 clade of *Alphaproteobacteria*, the *Flavobacteriaceae* NS4 and NS5 marine groups, and marine cyanobacterium *Synechococcus* CC9902 (Figure 6). Marine-associated taxa generally decreased in relative abundance at sites located nearest the SADB WTP outfall.

### Relationship Between SourceTracker, Human Marker, and *Enterococcus* Results

**FIGURE 7:**
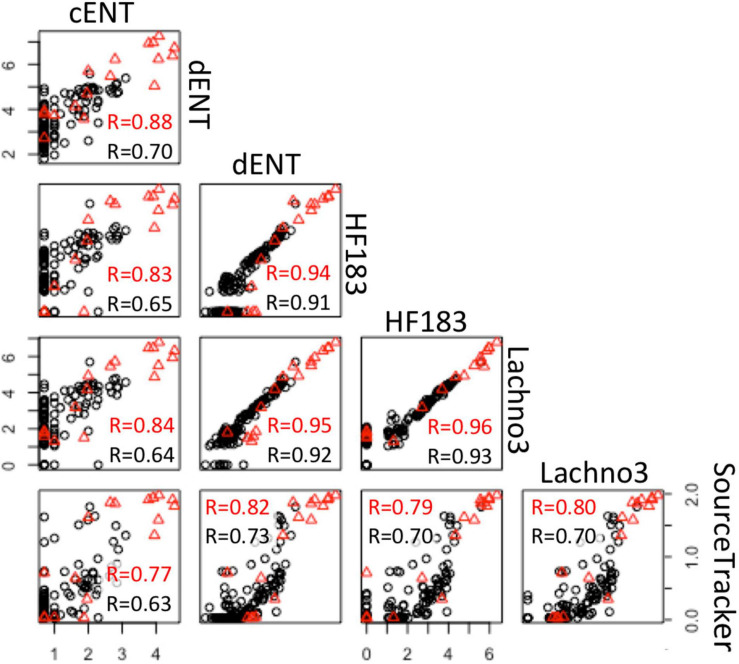
Correlations between log-normalized microbial indicator (enterococci by culture and ddPCR and human markers, HF183 and Lachno3) results and log-normalized (x + 1) SADB WTP contributions estimated by SourceTracker. Red *r*-values refer to correlations between data collected at all sites. Black *r*-values refer to correlations between environmental beach sites only (with the SADB WTP outfall and mixing zone excluded).

There were statistically significant positive correlations (*p* < 0.05) between the magnitude of the SADB WTP contribution determined by the SourceTracker algorithm and human marker measurements, when SADB WTP was included as the only contamination source. Human marker levels, Lachno3, and HF183, were strongly correlated with estimated percent contribution from the SADB WTP outfall, *r* = 0.80 and *r* = 0.70, respectively (Figure 7).

Significant correlations were present between enterococci and human marker measurements (HF183 and Lachno3), regardless of measurement method (all *r* > 0.6, all *p*-values < 0.01; Figure 7). Human marker measurements, Lachno3 versus HF183, were also highly correlated to each other (*r* = 0.96, *p* < 0.001; Figure 7), as were *Enterococcus* levels by culture and digital PCR (*r* = 0.88, *p* < 0.001; Figure 7).

## Discussion

### Alongshore Human Fecal Contamination Trends

The SADB WTP discharge at Punta Bandera represents a consistent point source of human fecal contamination to the nearshore environment, with the geographic extent of its impact dependent on dilution and dispersion processes (Kim et al., 2009). In this study, the use of a molecular toolbox approach allowed for an increased understanding of how the SADB plume impacts water quality in the United States/Mexico border region. Digital PCR analysis of human-associated markers and 16S rRNA bacterial community sequencing both identified a significant gradient from the SADB WTP outfall at Punta Bandera moving south to north, when current direction was from the south, with sites closer to the discharge impacted more heavily.

Previous tracer studies have found that nearshore freshwater sources can either be rapidly transported offshore or become entrained within the surf zone, depending on wave conditions and source volume and flow rates (Rodriguez et al., 2018). During this study, conditions observed during Event 3 and Event 4 reflect the significant impact changing ocean condition can have on the impact of the SADB plume within the nearshore coastal zone. The strongest gradient in human fecal contamination (by SourceTracker and human-associated marker) was observed during Event 4, which targeted a longer south swell event. Swell direction was coming from the south for several days preceding sampling, which likely contributed to the higher human-associated marker concentrations and the more limited dilution of the plume observed. During this event, SourceTracker analysis attributed between 1 and 5% of total beach water at sites in San Diego, CA, United States and between 5 and 50% of beach water at popular beach sites located in Tijuana, Mexico to the SADB WTP. Considering that the SADB WTP outfall consists of minimally treated sewage, exposure to the plume has the potential for significant health risk. The results observed during Event 4 suggest that the impact of the SADB WTP plume can extend to beaches far upcoast, with evidence of the plume detected up to 20 km north of the SADB WTP outfall at Punta Bandera. These results support previous modeling efforts conducted that have illustrated that the SADB WTP plume can propagate northward during south swell events, leading to minimally treated sewage transported to San Diego beaches as far upcoast as 32 km, under the right conditions (Feddersen et al., 2020).

In contrast to the far-reaching extent of the plume during Event 4, during the first 2 days of Event 3, when currents were coming from the west, human associated markers were either not detected or were only present near the limit of detection at sites upcoast of the SADB WTP outfall. SourceTracker analysis attributed 1–2% of total beach water to the SADB WTP at sites located to the north of the plant in Tijuana, Mexico and 1–6% of total beach water to the plume at site MX1, located <1 mile south of the SADB WTP outfall. Although not the focus of this study, these results suggest that under certain ocean conditions the plume may be quickly transported offshore, having a lesser impact on beach water quality.

Further bolstering the SourceTracker results, the abundant taxa in the microbial communities revealed a distinct sewage plume assemblage that contrasted with the coastal ocean assemblage. *Acidovorax, Bacteroides, Cloacibacterium*, *Comamonas*, *Macellibacteroides*, and potentially pathogenic genera *Acinetobacter*, *Aeromonas*, and *Arcobacter*, the most abundant taxa in this sewage plume assemblage, have been consistently reported to be abundant taxa in sewage 16S rRNA microbiomes throughout the world (e.g., McLellan et al., 2010; Vandewalle et al., 2012; Shanks et al., 2013; Cai et al., 2014; Newton et al., 2015; Ahmed et al., 2017; Numberger et al., 2019). In contrast, the most abundant taxa identified in the coastal ocean assemblage were typical of the environmental marine microbiome and have all been identified previously as abundant in southern California coastal ocean communities: *Alphaproteobacteria* including the ubiquitous SAR11 clade, *Amylibacter*, *Planktomarina*, *Planktotalea*, and the coastal HIMB11 *Roseobacter* group, *Flavobacteriaceae* marine groups NS4 and NS5, and *Synechococcus* CC9902, a ubiquitous marine cyanobacterium (Morris et al., 2002; Fuhrman et al., 2006; Dufresne et al., 2008; Giebel et al., 2009; Steele et al., 2011; Cram et al., 2015). The relative abundances of taxa associated with the sewage plume assemblage decreased with increasing distance from the SADB WTP outfall and at the same time, the coastal ocean assemblage became much more abundant, matching the SourceTracker results. The clear difference between the communities, along with the spatial gradient from the outfall, provides another line of evidence illustrating the influence of the SADB WTP plume during south swells.

Paired surf zone samples were also collected for comparison to the shoreline samples during the last two south swell events. There were no significant differences between the surf zone and shoreline samples collected and results from the two sampling areas were highly correlated, suggesting that under the water quality conditions measured in this study, shoreline samples collected as part of routine monitoring efforts are likely to reflect water quality conditions in the surf zone. This is contrast to previous efforts that found higher concentrations of FIB in the shoreline versus surf zone (SCCWRP, 2007). However, locations tested during those efforts were all located in close proximity to flowing freshwater creek inputs.

### Human Source Identification Limitations

It was originally hypothesized that the effect of treatment processes at the SADB WTP may be large enough to alter the bacterial community of the wastewater (Hu et al., 2012), allowing for discrimination between contamination from the SADB WTP and wastewater traveling to beach sites from the Tijuana River. However, neither detection of human-associated markers by ddPCR or SourceTracker analysis of 16S rRNA sequences were able to definitively differentiate between human fecal contamination from these two sources, despite treatment and transport differences. These two primary sources harbored similar bacterial communities, with variations observed only for bacterial taxa that were present at low abundances. This is perhaps not surprising, as it is well documented that the majority of fecal contamination to the Tijuana River emanates from faulty infrastructure in the City of Tijuana and that a portion of the sewage that spills into the river is also diverted directly to the SADB WTP (USEPA, 2014). Moving forward, taxa more closely associated with the SADB outfall could be targeted for specific marker analysis that may provide better discrimination between the SADB WTP and similar human sources, like the Tijuana River.

Overall, results obtained using human-associated markers and SourceTracker analysis of 16S rRNA sequences were generally in agreement and positively correlated with only slight differences observed in estimated dilution of the SADB WTP plume by the two methods. These differences may be attributed to differences in environmental fate and transport. The SourceTracker algorithm utilizes a combination of different sequences to create a unique fingerprint for each source. In contrast, PCR marker-based methods target a specific sequence associated with a particular source. Previous efforts have evaluated the persistence of both human markers (Bae and Wuertz, 2009; Ahmed et al., 2014; Mattioli et al., 2017) and wastewater associated bacterial communities in marine waters (Sassoubre et al., 2015; Zhang et al., 2015; Ahmed et al., 2018). Side-by-side comparisons are also needed that evaluate decay of these signals under relevant environmental conditions and to ensure the stability of bacterial community-based pollution assignments.

### Implications for Pollutant Tracking

The tools applied in this study represent a promising approach for further understanding pollution fate and transport in complex environmental settings. Modeling efforts have demonstrated the potential for the discharge plume from the SADB WTP to track north, across the United States/Mexico border under specific ocean conditions (CH2M Hill, 2009; Feddersen et al., 2020). However, this hypothesis has not been confirmed due to logistical challenges associated with both sampling in this region and with teasing apart multiple fecal pollution sources in the nearshore environment. In this study, more sensitive tools coupled with a targeted sampling approach that took advantage of specific ocean conditions helped characterize the potential impact of the SADB WTP plume. Although additional sources within this region could also be contributing to the elevated human marker levels; the trends observed in both the SourceTracker analysis and human-associated marker results tracked each other and swell conditions closely, pointing to the SADB WTP plume as a potential source of human fecal contamination to beach waters extending north, across the United States/Mexico border, under south swell conditions.

This is one of the first studies to effectively use microbial source tracking tools for nearshore plume characterization, which unlike other tracers, offer biological measurements that are a function of both decay and dilution. Hydrodynamic modeling-based approaches commonly used to predict plume transport typically rely on dyes, or other conservative tracers, to estimate how specific water sources move within the coastal environment (McCarthy et al., 2017). However, as pollutant sources travel in the environment, physical, biological, and chemical factors all govern the fate and transport of fecal-borne microbes (Sinton et al., 2002; Boehm et al., 2005, 2009; Rippy et al., 2013). FIB measurements have also been used to develop nearshore (Stark et al., 2016) and estuarine hydrodynamic models (Gao et al., 2015). However, FIB can come from multiple animal and human sources, confounding the ability to track specific pollutant sources. Advances in microbial source tracking tools may be a useful addition to hydrodynamic simulations, helping to contextualize, validate, and calibrate measurements made.

## Data Availability Statement

The datasets presented in this study can be found in online repositories. The names of the repository/repositories and accession number(s) can be found below: https://www.ncbi.nlm.nih.gov/, SRP250574.

## Author Contributions

AZ-F, JS, JG, and MD contributed to conception and design of the study. AZ-F, MG, XX, CS, and MS contributed to sample and statistical analyses. CS, MS, XX, and AZ-F contributed to data interpretation. AZ-F wrote the first draft of the manuscript. All authors contributed to revision, read, and approved the submitted version.

## Conflict of Interest

The authors declare that the research was conducted in the absence of any commercial or financial relationships that could be construed as a potential conflict of interest.

## Publisher’s Note

All claims expressed in this article are solely those of the authors and do not necessarily represent those of their affiliated organizations, or those of the publisher, the editors and the reviewers. Any product that may be evaluated in this article, or claim that may be made by its manufacturer, is not guaranteed or endorsed by the publisher.

## Footnotes

1
http://cdip.ucsd.edu/

2
https://tidesandcurrents.noaa.gov/

## Supplementary Material

Supplementary Table 1 Description of number of samples collected at each site and events when sampling occurred at each site.

Supplementary Table 2 Primer and probe sequences utilized for microbial source tracking assays.
