# Supplementary material for: A Combined Digital PCR and Next Generation DNA-Sequencing Based Approach for Tracking Nearshore Pollutant Dynamics Along the Southwest United States/Mexico Border
Source: Front Microbiol. 2021 Aug 6;12:674214. doi: 10.3389/fmicb.2021.674214 (PMC8377738; doi:10.3389/fmicb.2021.674214)
Supplement: Supplementary Table 1 — Description of number of samples collected at each site and events when sampling occurred at each site. [file Table_1.docx]

**Supplemental Material**

**Table S1**. Description of number of samples collected at each site and events when sampling occurred at each site.

| **Site** | **Local** | **Events Sampled** | **# Days Total** |
| --- | --- | --- | --- |
| MX1 | Swashzone | 1, 2, 3, 4 | 11 |
| MX2 | Swashzone | 1, 2, 3, 4 | 11 |
| MX3 | Swashzone | 1, 2, 3, 4 | 11 |
| MX4 | Swashzone | 1, 2, 3, 4 | 11 |
| MX5 | Swashzone | 2, 3, 4 | 9 |
| SD1 | Swashzone | 1, 2, 3, 4 | 11 |
| SD1 | Surfzone | 3, 4 | 5 |
| SD2 | Swashzone | 1, 2, 3, 4 | 11 |
| TJRM | Swashzone | 3, 4 | 6 |
| TJRM | Surfzone | 3, 4 | 5 |
| SD3 | Swashzone | 1, 2, 3, 4 | 11 |
| SD3 | Surfzone | 3, 4 | 5 |
| IB1 | Swashzone | 3, 4 | 6 |
| IB1 | Surfzone | 3, 4 | 5 |
| IB2 | Swashzone | 3, 4 | 6 |
| IB2 | Surfzone | 3, 4 | 5 |
| SD4 | Swashzone | 1, 2, 3, 4 | 11 |
| SD4 | Surfzone | 3, 4 | 5 |
| SD5 | Swashzone | 1, 2, 3, 4 | 11 |
| SD5 | Surfzone | 3, 4 | 5 |

**Table S2**. Primer and probe sequences utilized for microbial source tracking assays.

| **Target** | **Gene** | **Primer/Probe** | **Sequences** | **Reference** |
| --- | --- | --- | --- | --- |
| Enterococcus | 23S rRNA | EnteroF1A | GAGAAATTCCAAACGAACTTG | USEPA, 2012; Cao et al. 2015 |
|  |  | EnteroR1 | CAGTGCTCTACCTCCATCATT |  |
|  |  | GPL813TQ | [FAM]-TGGTTCTCTCCGAAATAGCTTTAGGGCTA-[BHQ1] |  |
| Human-associated Bacteroidales (HF183) | 16S rRNA | HF183ND | ATCATGAGTTCACATGTCCG | Cao et al. 2015 |
|  |  | BthetR1 | CGTAGGAGTTTGGACCGTGT |  |
|  |  | BthetP1 | [HEX]-CTGAGAGGAAGGTCCCCCACATTGGA-[BHQ1] |  |
| Human-associated Lachnospiraceae (Lachno3) | 16S rRNA | Lachno3F | CAACGCGAAGAACCTTACCAAA | Feng et al. 2018 |
|  |  | Lachno3R | CCCAGAGTGCCCACCTTAAAT |  |
|  |  | Lachno3P | [FAM]-CTCTGACCGGTCTTTAATCGGA-[MGB] |  |
| Halophile (N. pharaonis) | gyrA | NPgyrAF | ACGATTACCTGCTCTGCTTTAC | SCCWRP, 2019 |
|  |  | NPgyrAR | CGTTGAGGTCGAGAACATTGA |  |
|  |  | NPgyrAP | [FAM]-CAAGGGCAGGTCTATCGGCTGAAG-[BHQ1] |  |

**Figure Captions**

**Figure S1.** Wave direction [top plot], significant wave height (SWH), and tidal conditions [bottom plot] during Event 1 sampling. Red lines indicate days when sampling occurred, with black lines reflecting conditions preceding and following sampling dates. Black arrow denotes Tijuana River flows, and magnitude in total million gallons (MG), that occurred near the sampling timeframe and were not diverted to collectors.

**Figure S2.** Wave direction [top plot], significant wave height (SWH), and tidal conditions [bottom plot] during Event 2 sampling. Red lines indicate days when sampling occurred, with black reflecting conditions preceding and following sampling dates. Black arrows denote Tijuana River flows, and magnitude in total million gallons (MG), that occurred near the sampling timeframe and were not diverted to collectors.

**Figure S3**. Wave direction [top plot], significant wave height (SWH), and tidal conditions [bottom plot] during Event 3 sampling. Red lines indicate days when sampling occurred, with black reflecting conditions preceding and following sampling dates. Black arrows denote Tijuana River flows, and magnitude in total million gallons (MG), that occurred near the sampling timeframe and were not diverted to collectors.

**Figure S4.** Wave direction [top plot], significant wave height (SWH), and tidal conditions [bottom plot] during Event 4 sampling. Red lines indicate days when sampling occurred, with black reflecting conditions preceding and following sampling dates.

**Figure S5**. Log reduction in enterococci by culture (cENT) and by ddPCR (dENT) and human markers (HF183 and Lachno3) concentrations at sites moving away from the SADB WTP outfall at Punta Bandera. The outfall is denoted on the figure as the red dashed line. All sampling days are shown with dots, with color representing sampling event. The boxplots represent all data combined, across the four sampling events. The black dashed like represents the location of the U.S./Mexico border.

**Figure S6.** Contributions from potential sources (SADB WTP and Tijuana River) in each sink site using SourceTracker. Sequencing data utilized was processed using DADA2. Average contributions represent duplicate samples from each site. Sites are arranged from a north to south along the x-axis. A) SADB WTP included as the only source. B) SADB WTP and Tijuana River included as potential sources.

**Figure S7.** Concentration of enterococci (by culture and ddPCR) and human marker (HF183 and Lachno3) for San Diego swashzone (white bars) versus surfzone (grey bars) sites, sampled during Event 3 and Event 4. Sites are in order from South to North on the x-axis.
