# Supplementary figures and images for: A Combined Digital PCR and Next Generation DNA-Sequencing Based Approach for Tracking Nearshore Pollutant Dynamics Along the Southwest United States/Mexico Border
Source: Front Microbiol. 2021 Aug 6;12:674214. doi: 10.3389/fmicb.2021.674214 (PMC8377738; doi:10.3389/fmicb.2021.674214)

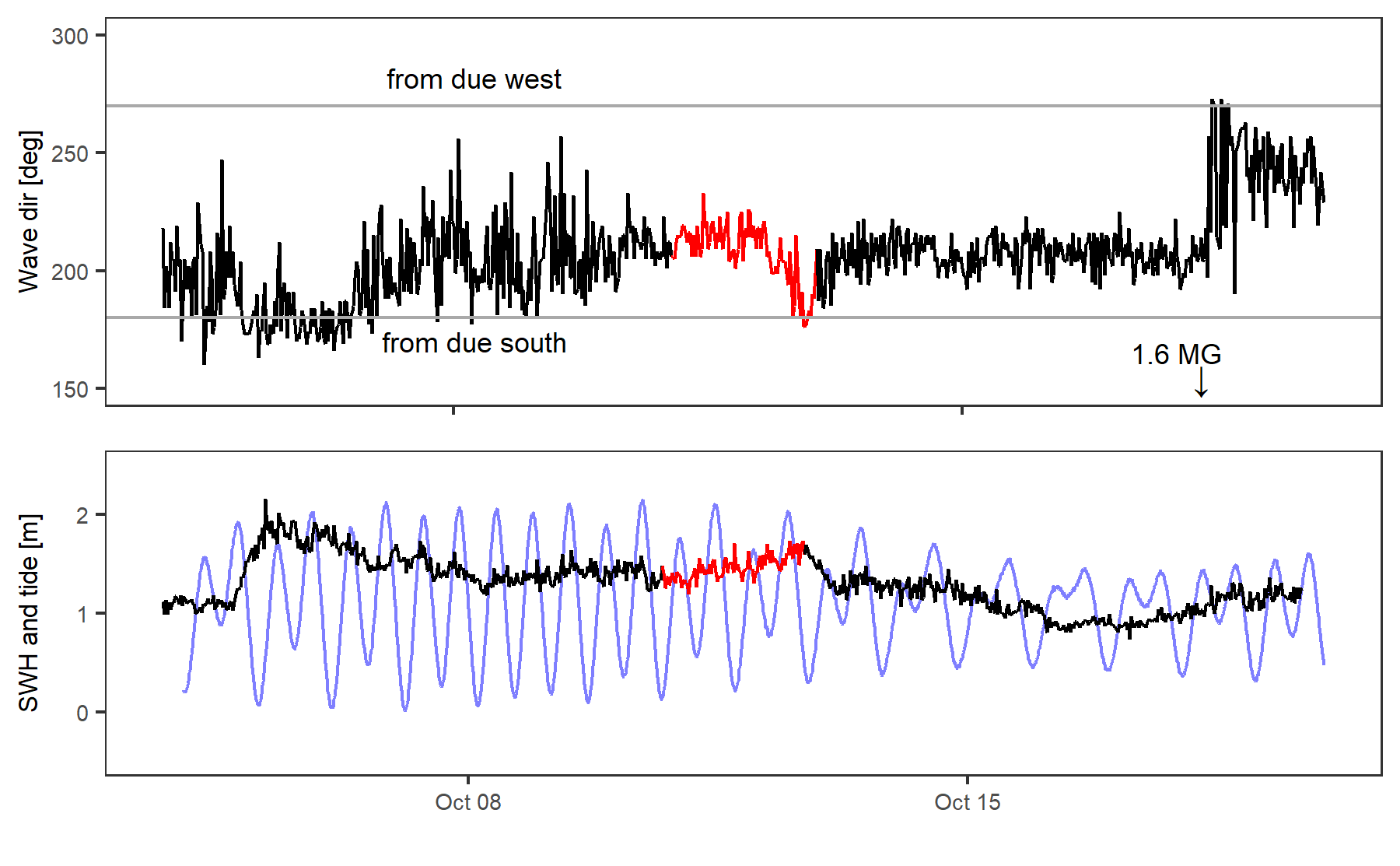

Supplement: Supplementary file 2 [file Image_1.PNG]

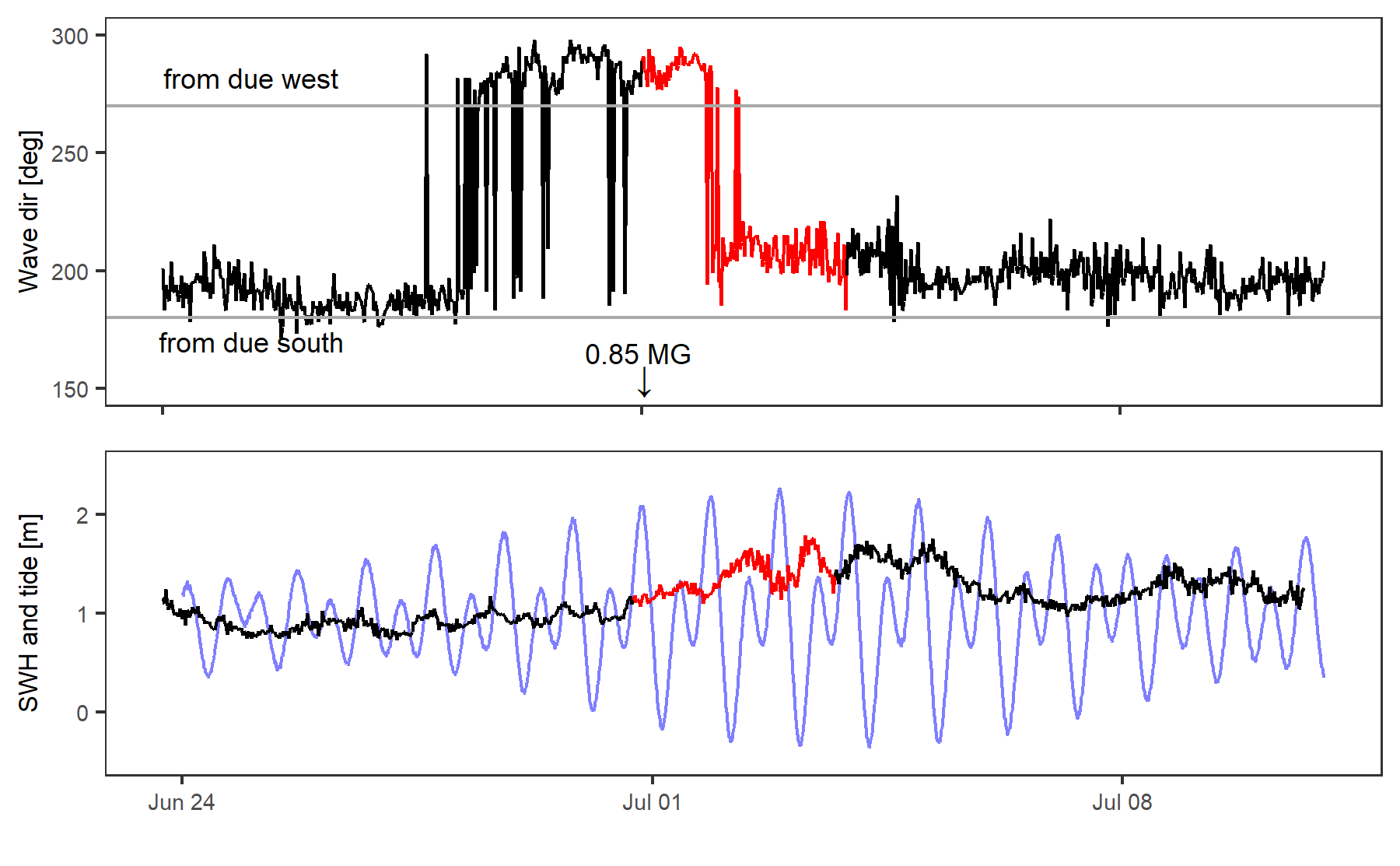

Supplement: Supplementary file 3 [file Image_2.PNG]

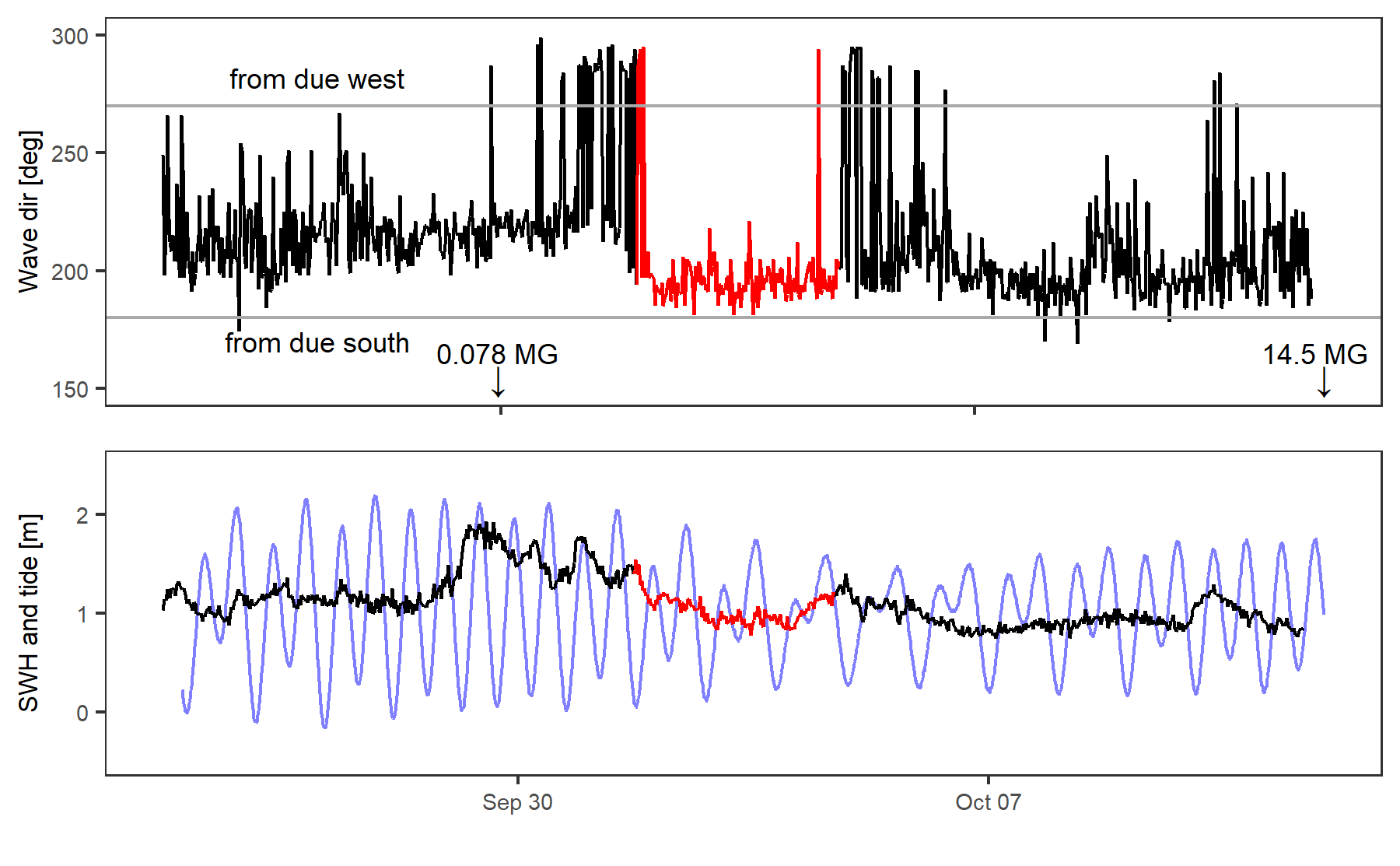

Supplement: Supplementary file 4 [file Image_3.PNG]

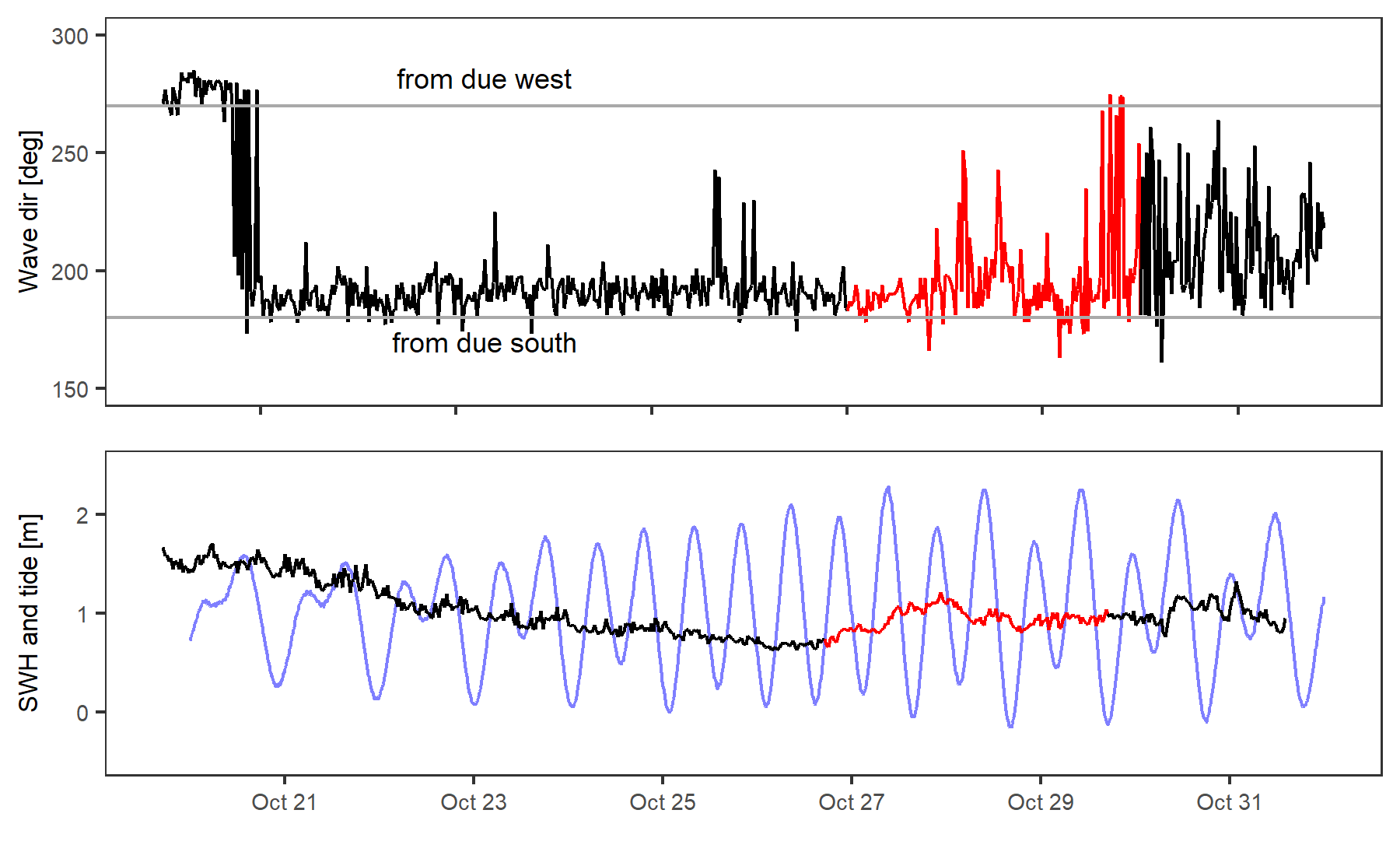

Supplement: Supplementary file 5 [file Image_4.PNG]

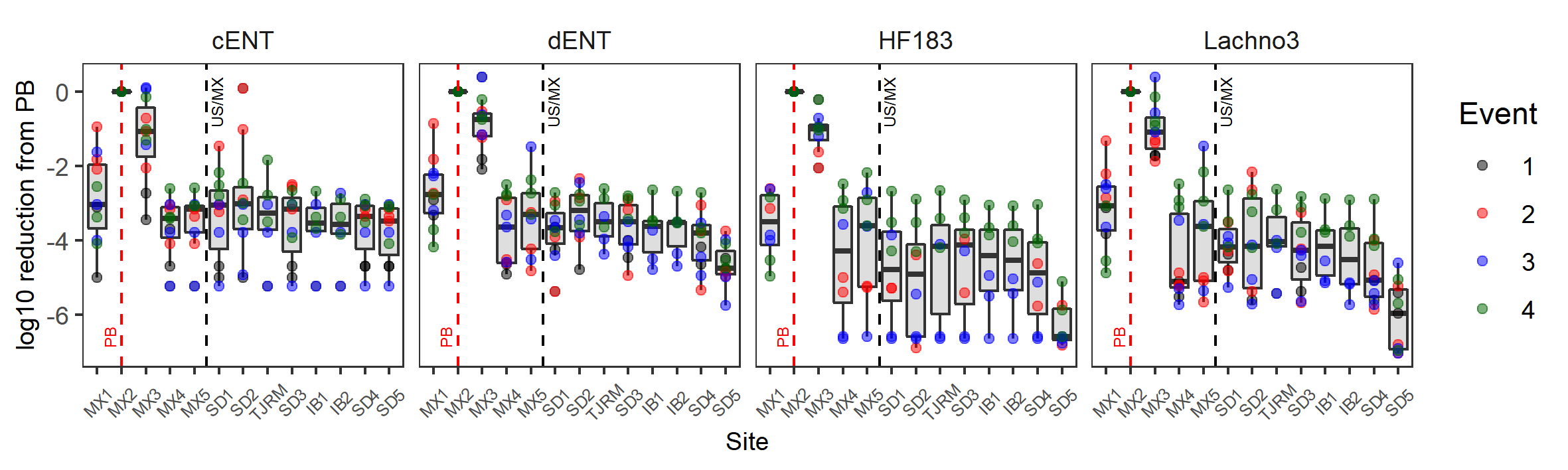

Supplement: Supplementary file 6 [file Image_5.TIFF]

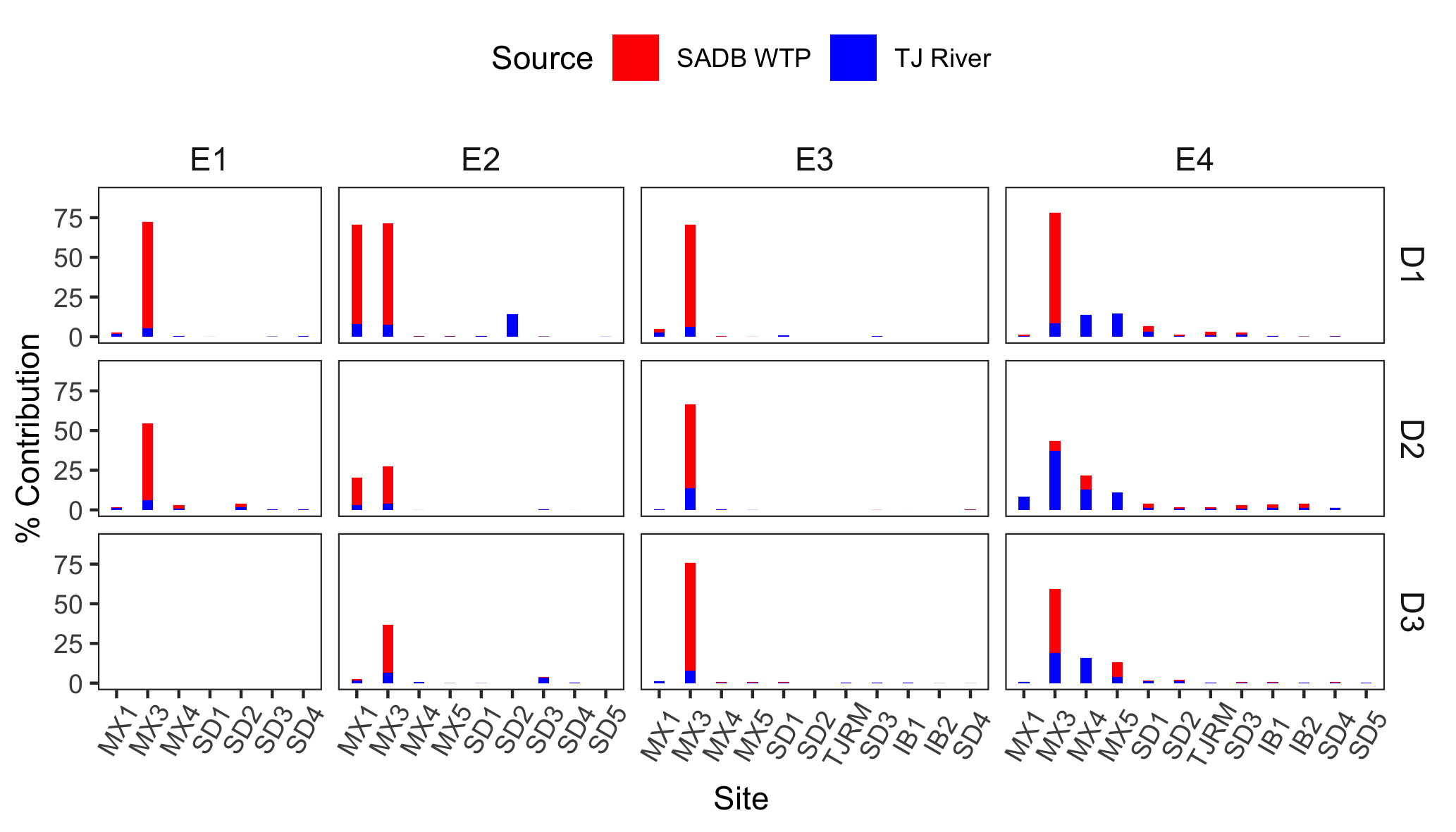

Supplement: Supplementary file 7 [file Image_6.TIFF]

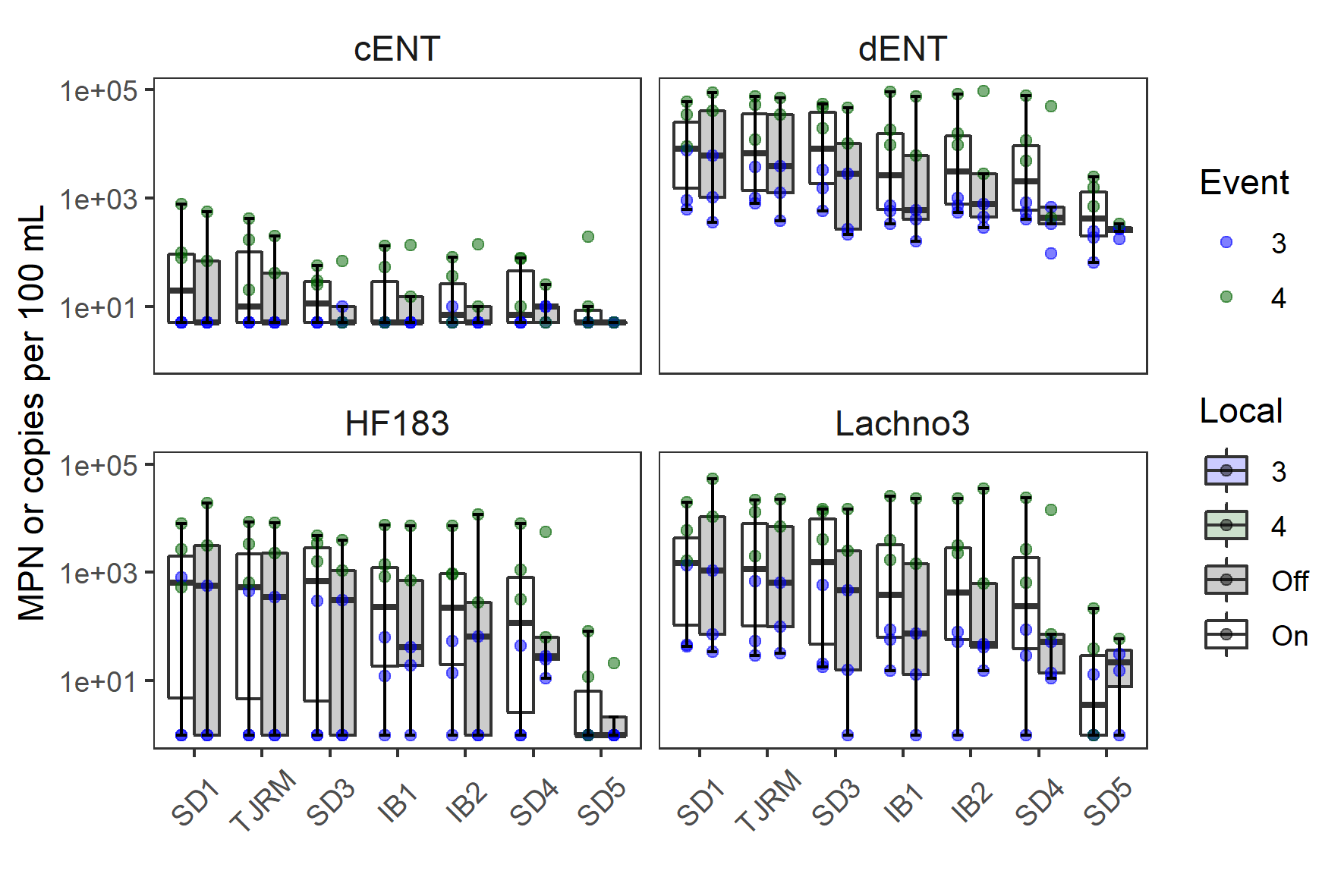

Supplement: Supplementary file 8 [file Image_7.TIFF]

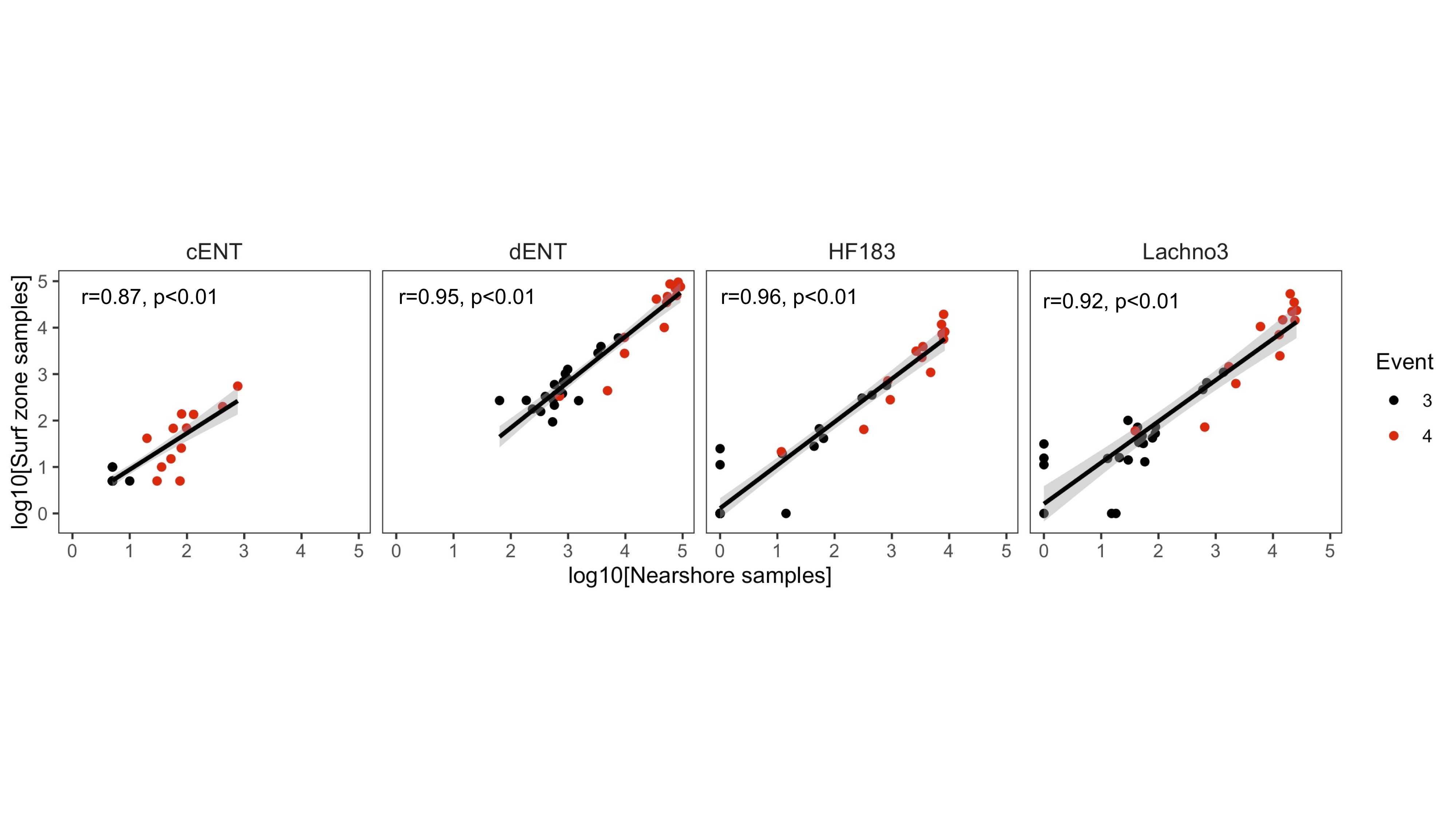

Supplement: Supplementary file 9 [file Image_8.TIFF]
